# Comparison of the Utility and Validity of Three Scoring Tools to Measure Skin Involvement in Patients With Juvenile Dermatomyositis

**DOI:** 10.1002/acr.22867

**Published:** 2016-09-16

**Authors:** Raquel Campanilho‐Marques, Beverley Almeida, Claire Deakin, Katie Arnold, Natacha Gallot, Maria de Iorio, Kiran Nistala, Clarissa A. Pilkington, Lucy R. Wedderburn, on behalf of the Juvenile Dermatomyositis Research Group, Kate Armon, Joe Ellis‐Gage, Holly Roper, Vanja Briggs, Joanna Watts, Liza McCann, Ian Roberts, Eileen Baildam, Louise Hanna, Olivia Lloyd, Phil Riley, Ann McGovern, Clive Ryder, Janis Scott, Beverley Thomas, Taunton Southwood, Eslam Al‐Abadi, Sue Wyatt, Gillian Jackson, Tania Amin, Mark Wood, Vanessa VanRooyen, Deborah Burton, Joyce Davidson, Janet Gardner‐Medwin, Neil Martin, Sue Ferguson, Liz Waxman, Michael Browne, Mark Friswell, Helen Foster, Alison Swift, Sharmila Jandial, Vicky Stevenson, Debbie Wade, Ethan Sen, Eve Smith, Lisa Qiao, Stuart Watson, Helen Venning, Rangaraj Satyapal, Elizabeth Stretton, Mary Jordan, Ellen Mosley, Anna Frost, Lindsay Crate, Kishore Warrier, Lucy Wedderburn, Clarissa Pilkington, Nathan Hasson, Kiran Nistala, Sue Maillard, Elizabeth Halkon, Virginia Brown, Audrey Juggins, Sally Smith, Sian Lunt, Elli Enayat, Hemlata Varsani, Laura Kassoumeri, Laura Beard, Katie Arnold, Yvonne Glackin, Stephanie Simou, Raquel Campanilho‐Marques, Beverley Almeida, Kevin Murray, John Ioannou, Linda Suffield, Muthana Al‐Obaidi, Helen Lee, Sam Leach, Helen Smith, Nick Wilkinson, Emma Inness, Eunice Kendall, David Mayers, Jacqui Clinch, Helen Pluess‐Hall

**Affiliations:** ^1^ University College London and Great Ormond Street Hospital for Children NHS Foundation TrustLondonUK; ^2^ University College LondonLondonUK; ^3^ Great Ormond Street Hospital for Children NHS Foundation TrustLondonUK; ^4^ Arthritis Research UK Centre for Adolescent Rheumatology, University College London, University College London Hospitals, and Great Ormond Street Hospital for Children NHS Foundation TrustLondonUK

## Abstract

**Objective:**

To compare the abbreviated Cutaneous Assessment Tool (CAT), Disease Activity Score (DAS), and Myositis Intention to Treat Activity Index (MITAX) and correlate them with the physician's 10‐cm skin visual analog scale (VAS) in order to define which tool best assesses skin disease in patients with juvenile dermatomyositis.

**Methods:**

A total of 71 patients recruited to the UK Juvenile Dermatomyositis Cohort and Biomarker Study were included and assessed for skin disease using the CAT, DAS, MITAX, and skin VAS. The Childhood Myositis Assessment Scale (CMAS), manual muscle testing of 8 groups (MMT8), muscle enzymes, inflammatory markers, and physician's global VAS were recorded. Relationships were evaluated using Spearman's correlations and predictors with linear regression. Interrater reliability was assessed using intraclass correlation coefficients.

**Results:**

All 3 tools showed correlation with the physician's global VAS and skin VAS, with DAS skin showing the strongest correlation with skin VAS. DAS skin and CAT activity were inversely correlated with CMAS and MMT8, but these correlations were moderate. No correlations were found between the skin tools and inflammatory markers or muscle enzymes. DAS skin and CAT were the quickest to complete (mean ± SD 0.68 ± 0.1 minutes and 0.63 ± 0.1 minutes, respectively).

**Conclusion:**

The 3 skin tools were quick and easy to use. The DAS skin correlated best with the skin VAS. The addition of CAT in a bivariate model containing the physician's global VAS was a statistically significant estimator of skin VAS score. We propose that there is scope for a new skin tool to be devised and tested, which takes into account the strengths of the 3 existing tools.

## INTRODUCTION

Juvenile dermatomyositis (DM) is a rare inflammatory disease of childhood that predominantly affects muscles and skin but is also a systemic multiorgan disease 1. It is the most common idiopathic inflammatory myopathy (IIM) of childhood, with a reported incidence of 2–3 new cases per million children per year 2. Juvenile DM is clinically heterogeneous, with some children experiencing mild disease while others display a more severe disease progression. Skin manifestations include Gottron's papules, erythema, heliotrope rash, ulceration, lipodystrophy, and calcinosis 3. Within the UK Juvenile Dermatomyositis Cohort and Biomarker Study, 88% of patients with juvenile DM have rash, 23% have skin ulceration, and 7% have calcinosis 4. Children can show severe myositis with or without skin disease, or with skin disease as the predominant feature and mild myositis. This variation poses a problem, since skin disease can be difficult to control and is less responsive to standard treatment than muscle disease. Evidence suggests that poorly controlled skin disease is a predictor of severity and damage 5. An accurate assessment of skin involvement is of critical importance in determining clinical status, making treatment decisions, and predicting outcome in juvenile DM.

Box 1 **Significance & Innovations**

- Current tools available to measure skin involvement in juvenile dermatomyositis (DM) are each different, and no single tool includes all aspects of skin involvement.
- All 3 skin tools correlated with physician's global assessment and a skin global assessment, and the tools correlate with each other.
- There is a need for standardized quantification of skin involvement in juvenile DM to inform treatment choices and compare patients between centers.

In clinical practice, muscle symptoms of juvenile DM are frequently the main initial focus. Formal measures such as the Childhood Myositis Assessment Scale (CMAS) 6 and manual muscle testing of 8 groups (MMT8) 7 exist to standardize the muscle assessment. Both are part of the agreed Juvenile Dermatomyositis Paediatric Rheumatology International Trials Organisation 8 and International Myositis Assessment and Clinical Studies Group 9 core disease activity measures. Despite the importance of skin disease, at present many physicians and other health care professionals who care for children with juvenile DM do not routinely measure the severity of skin activity using a validated tool. Several tools have been proposed to measure skin disease in juvenile DM. These include the abbreviated Cutaneous Assessment Tool (CAT), encompassing active skin disease and skin damage 10, the Disease Activity Score (DAS) 11, and the Myositis Intention to Treat Activity Index (MITAX) 12. The latter 2 tools both have skin components (although they are not skin specific).

Only 3 specific items are in all 3 tools (Gottron's papules, heliotrope rash, and periungual capillary changes). The MITAX was designed to assess all organ systems likely to be involved in IIMs and is not specific to skin alone. The MITAX does not include some cutaneous clinical features such as calcinosis and lipoatrophy, which are felt to be severe skin manifestations by many physicians. It consists of a relatively long form with a complex scoring system that then requires conversion to a categorical scale. The reliability of the MITAX has been found to be fair to good for most aspects of the tool in initial testing 12. The DAS has good reliability and validity 11. Like the MITAX, it was not solely designed to test the skin alone. It is easy to use, but the weighting of the items is relatively arbitrary. It also does not take into account the severity or different forms of some signs, such as different types of vasculitic changes, and does not consider several items, including calcinosis, lipoatrophy, shawl sign, V sign, mechanic's hands, or ulceration. The abbreviated CAT score has been shown to have consistency, nonredundancy, and good construct validity 10. It was designed specifically to assess skin disease in juvenile DM. This tool is the easiest to use (items are scored dichotomously: the indicator is present or not), and it is the tool that considers the most skin features. The following items that are scored in the CAT are not included in the other 2 tools: linear extensor erythema, shawl sign, V sign, non‐sun exposed erythema, livedo reticularis, cuticular overgrowth, subcutaneous edema, calcinosis, and lipoatrophy. However, it gives equal weighting to all skin signs, whereas in clinical practice many physicians may deem some signs, such as ulceration to be more important than, for example, livedo reticularis, or hypopigmented Gottron's papules. In addition, the CAT does not consider the distribution of skin involvement.

However the optimal tool is unknown. The goal of this study was to directly compare these 3 tools and evaluate the measurement characteristics of the CAT, DAS, and MITAX, including construct validity, interrater reliability, and their ability to detect skin disease activity in a large cohort of children and young people with juvenile DM, in order to define which existing tool best assesses skin disease in juvenile DM.

## PATIENTS AND METHODS

#### Patients and measures

A total of 71 patients were recruited via the UK Juvenile Dermatomyositis Cohort and Biomarker Study. All met the Bohan and Peter classification criteria 13, 14 for definite or probable juvenile DM and were ages <16 years at the time of diagnosis. At clinical assessment, a structured history and physical examination were obtained. A complete skin assessment was performed by 2 pediatric rheumatologists (RC‐M, BA), both trained by specialists with expertise in juvenile DM in a tertiary pediatric rheumatology center. Each patient was assessed for skin disease using the abbreviated CAT, DAS, MITAX, and an overall 0–10‐cm physician's skin visual analog scale (VAS), considering both activity and damage. A subset of patients (n = 20) was assessed at 2 time points, at a mean ± SD time of 5.52 ± 7.08 months apart. The abbreviated CAT binary method uses 21 items, subdividing skin involvement into active disease and disease damage lesions, with 17 items in the active section and 11 in the damage section. Each lesion receives a score of 1 if present and 0 if absent 10. Higher scores indicate greater skin disease activity. The DAS is a 20‐point scale, with 2 subsections, 1 assessing skin disease (range 0–9) and the second assessing muscle inflammation (range 0–11). Higher scores indicate greater disease activity 11. The MITAX 12 was developed along similar lines to the British Isles Lupus Assessment Group score, used to assess disease activity in patients with lupus. The scoring system is based on the physician's intention to treat. The MITAX assesses the following organ systems: constitutional, cutaneous, skeletal, gastrointestinal, pulmonary, cardiac, and musculoskeletal. Each item is scored between 0 and 4 (based on the presence of clinical features at the time or symptoms within the previous 4 weeks). For each of the organ system sections, the scale is categorical, with scores of 0, 1, 3, or 9. For the DAS and MITAX, both tools were scored in their entirety. For the purposes of this study the skin section was the area of interest for both these tools.

Core outcome variables for juvenile DM were collected, including CMAS (6), MMT8 (7), the physician's global assessment of overall disease activity (using a 0–10‐cm VAS), serum levels of muscle‐associated enzymes (creatine kinase [CK; units/liter], aspartate aminotransferase [AST; units/liter], alanine transaminase [ALT; units/liter] and lactate dehydrogenase [LDH; units/liter]) and inflammatory markers (C‐reactive protein level [CRP; mg/liter] and erythrocyte sedimentation rate [ESR; mm/hour]).

#### Statistical analysis

Nonparametric and parametric summary statistics were used to describe the distribution of score data for each tool. Validity was assessed by comparing the tools with the skin VAS as a means of identifying whether they were clinically and accurately reflecting current skin disease states. Construct validity determines whether a variable (or set of variables), in this case the skin tools, is related to other measures and do indeed measure skin disease. As there is no accepted gold standard for assessing skin disease in juvenile DM, the skin VAS was used to test whether the 3 outcome skin tools accurately reflected their purported outcome measurements. Construct validity also examines the relationships between the tools and measures of other constructs, such as laboratory results and muscle testing (CMAS and MMT8). The nonparametric Spearman's rank correlation (r_s_) was selected for analysis of correlation between each score tool and skin VAS, since the distributions of scores generated using these tools were not normal and also because the MITAX skin score is not measured on a linear scale. For the purpose of this analysis, correlations ≥0.75 (and conversely ≤ − 0.75) were considered strong, correlations ranging from 0.40 to 0.75 (and conversely − 0.4 to − 0.75) were considered moderate, and correlations <0.4 (and conversely > − 0.4) were considered poor. To correct for multiple comparisons, a 2‐sided *P* value less than 0.001 was considered statistically significant.

The relative ability of the skin tools to predict skin VAS was assessed by linear regression modelling. Univariate models were compared by considering the proportion of variance in skin VAS explained by the model, as estimated by the adjusted model R^2^. Bivariate models were used to assess which combinations of measures contributed the most to explain the variance in skin VAS. Bivariate models were compared to their respective nested univariate models using analysis of variance (ANOVA).

The variance inflation factor (VIF) was calculated to detect possible multicollinearity between variables in the bivariate models. For patients whose skin disease was assessed at multiple time points, sensitivity of each tool to measure change over time was estimated by calculating the standardized response mean (SRM). The SRM represents the mean of the differences in the tools between time points, divided by the SD of those differences. The SRM was calculated for the tools that are measured on a linear scale (skin VAS, DAS skin, and CAT activity). Absolute numbers were used for the ranges. Cohen's thresholds 15 (trivial <0.20, small ≥0.20 to <0.50, moderate ≥0.50 to <0.80, or large ≥0.80) were used for grading the SRM values.

To assess interrater reliability, 18 of the 71 patients were assessed by the 2 pediatric rheumatologists on the same day; each was unaware of the other's scores. Agreement intraclass correlation coefficients were calculated for the overall score of each tool to assess how consistent the scores were between the 2 physicians. This calculation was performed using a 2‐way model to account for the variability attributable to observers in addition to the variability attributable to the subjects. A value >0.75 was indicative of excellent agreement and between 0.4 and 0.75 was considered fair to good agreement 16. There was no disagreement between the physicians. Analyses were performed using R software, version 3.0.1. As multiple tests were used, 2‐sided *P* values less than 0.001 were considered to be statistically significant.

## RESULTS

#### Patients and outcome measures

**Figure 1:**
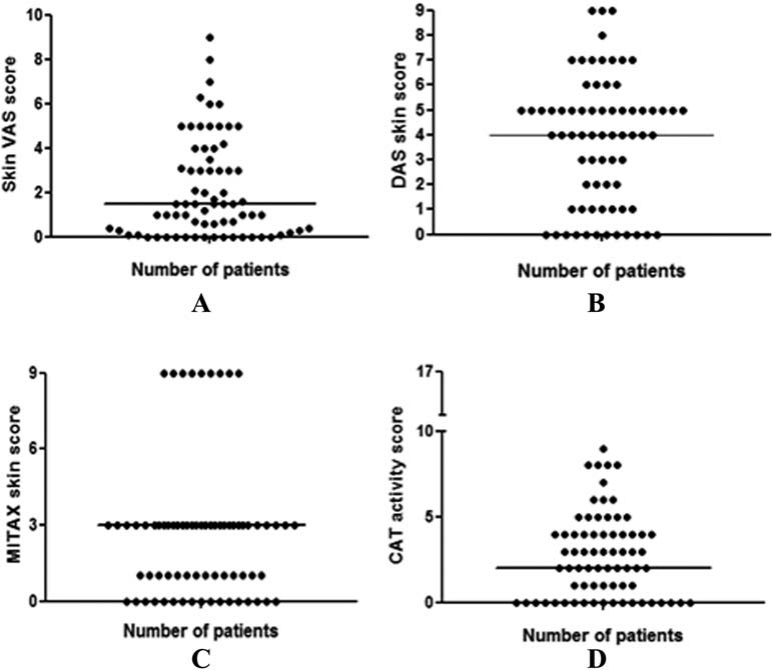
Frequency of skin scores in the 71 patients according to the skin tools. The panels show the scores for **A,** skin visual analog scale (VAS), **B,** Disease Activity Score (DAS) skin, **C,** Myositis Intention to Treat Activity Index (MITAX) skin, and **D,** Cutaneous Assessment Tool (CAT) activity. Each symbol represents a patient; horizontal lines represent the median.

Of the 71 patients assessed, 59.2% were female and 71.8% were white. At the time of assessment the mean ± SD age was 9.8 ± 3.8 years. The mean ± SD age at diagnosis was 6.6 ± 3.4 years, at assessment 9.8 ± 3.8 years, and duration at time of assessment 3.3 ± 3.0 years (Table 1). Our cohort had similar demographics to previously described cohorts 2 and is representative of juvenile DM patients. The disease activity measures at the time of assessment are shown in Table 2. The median skin VAS score at assessment was 1.5 (interquartile range 0.2–3.5). The median scores for the tools erred towards minimal to moderate evidence of skin disease. (Figure 1). The CMAS and MMT8 score medians demonstrated inactive or minimal muscle disease at the time of assessment.

**Table 1:** Demographic data of all patients^a^

Variables	Juvenile DM (n = 71)
Demographics	
Female	42 (59)
White	51 (72)
Age, mean ± SD years	9.8 ± 3.8
Age at diagnosis, mean ± SD years	6.0 ± 3.4
Disease duration, mean ± SD years	3.3 ± 3
Treatment at time of assessment	
PDN alone	2 (3)
MTX alone	20 (28.2)
MTX + PDN alone	20 (28.2)
AZA + PDN alone	5 (7)
MTX + IvIg alone	4 (5.6)
MTX + CYC alone	4 (5.6)
MTX + IvIg + CYC	4 (5.6)
Steroids + MTX + AZA	4 (5.6)
Biologic agents with other DMARDs	8 (11.2)

a Values are the number (%) unless indicated otherwise. DM = dermatomyositis; PDN = prednisolone; MTX = methotrexate; AZA = azathioprine; IvIg = intravenous immunoglobulin; CYC = cyclophosphamide; DMARDs = disease‐modifying antirheumatic drugs.

**Table 2:** Descriptive statistics of tools and disease activity measures^a^

Variables	No.	Median (IQR)
Tools		
CAT activity (range 0–17)	71	2 (0–4)
CAT damage (range 0–11)	71	0 (0–1)
DAS total (range 0–20)	71	5 (2–8)
DAS skin (range 0–9)	71	4 (1–5)
MITAX total (range 0–63)	71	3 (1–7)
MITAX skin (range 0–9)	71	3 (1–3)
Physician's evaluations		
Global VAS (range 0–10)	71	2.0 (0.1–4.0)
Skin VAS (range 0–10)	71	1.5 (0.2–3.5)
Muscle disease measures		
CMAS (range 0–53)	71	50 (46–53)
MMT8 (range 0–80)	71	80 (69–80)
CK (normal <150 units/liter)	55	89 (68–161)
Inflammatory markers		
CRP (normal <20 mg/liter)	57	5 (5–5)
ESR (normal <10 mm/hour)	55	9 (4–16)

a IQR = interquartile range; CAT = Cutaneous Assessment Tool; DAS = Disease Activity Score; MITAX = Myositis Intention to Treat Activity Index; VAS = visual analog scale; CMAS = Childhood Myositis Assessment Scale; MMT8 = manual muscle testing in 8 groups; CK = creatine kinase; CRP = C‐reactive protein; ESR = erythrocyte sedimentation rate.

#### Construct validity

**Figure 2:**
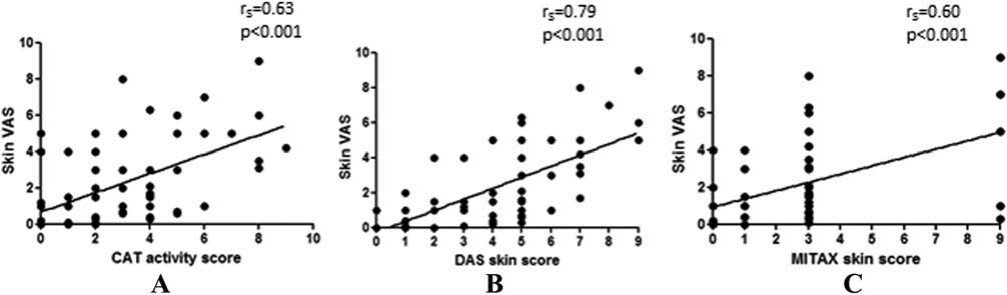
Analysis of relationships between skin visual analog scale (VAS) and skin tool scores. **A,** Cutaneous Assessment Tool (CAT) activity score, **B,** Disease Activity Score (DAS) skin, and **C,** Myositis Intention to Treat Activity Index (MITAX) skin score, with superimposed regression lines.

Spearman's correlation coefficients were used to assess construct validity of the skin tools by correlating each tool with either the skin VAS or physician's global VAS (Table 3). All skin assessment tools correlated positively with both the physician's global VAS and skin VAS. The CAT and the skin sections of both the DAS and MITAX correlated well with the skin VAS (r_s_ = 0.63, *P* < 0.001; r_s_ = 0.79, *P* < 0.001; and r_s_ = 0.60, *P* < 0.001, respectively) (Figure 2). The skin section of the DAS had a statistically higher correlation with the skin VAS than the other 2 tools. For the physician's global VAS, the correlation coefficient was strongest (r_s_ > 0.75) between physician's global VAS/total MITAX and physician's global VAS/total DAS, as all of these perform a generalized assessment of the disease.

**Table 3:** Correlation between the tools and other disease activity measures using Spearman's correlation with corresponding *P* value^a^

	CAT activity		CAT damage		DAS total		DAS skin		MITAX total		MITAX skin	
Disease activity measures	Values	*P*	Values	*P*	Values	*P*	Values	*P*	Values	*P*	Values	*P*
Physician's evaluations												
Skin VAS (n = 71)	0.63	< 0.001	0.34	0.004	0.74	< 0.001	0.79	< 0.001	0.60	< 0.001	0.60	< 0.001
Global VAS (n = 71)	0.59	< 0.001	0.19	0.11	0.78	< 0.001	0.68	< 0.001	0.79	< 0.001	0.67	< 0.001
Muscle disease measures												
CMAS (n = 71)	−0.42	< 0.001	0.2	0.1	−0.63	< 0.001	−0.42	< 0.001	−0.55	< 0.001	−0.35	0.003
MMT8 (n = 71)	−0.39	0.001	0.16	0.18	−0.66	< 0.001	−0.4	< 0.001	−0.6	< 0.001	−0.41	< 0.001
CK (n = 55)	0.13	0.33	−0.04	0.79	0.27	0.05	0.2	0.14	0.2	0.13	0.13	0.33
ALT (n = 53)	−0.09	0.46	0.02	0.88	0.01	0.96	0.02	0.88	−0.06	0.6	0.06	0.63
AST (n = 27)	0.09	0.46	−0.1	0.38	0.2	0.1	0.18	0.14	0.14	0.26	0.13	0.29
LDH (n = 48)	0.07	0.57	0.02	0.88	0.12	0.32	0.15	0.21	0.02	0.87	0.15	0.21
Inflammatory markers												
CRP (n = 57)	0.28	0.03	0.02	0.87	0.34	0.01	0.3	0.02	0.26	< 0.001	0.18	0.19
ESR (n = 55)	0.16	0.24	−0.11	0.94	0.36	0.01	0.32	0.02	0.38	0.005	0.28	0.04

a CAT = Cutaneous Assessment Tool; DAS = Disease Activity Score; MITAX = Myositis Intention to Treat Activity Index; VAS = visual analog scale; CMAS = Childhood Myositis Assessment Scale; MMT8 = manual muscle testing in 8 groups; CK = creatine kinase; ALT = alanine transaminase; AST = aspartate aminotransferase; LDH = lactate dehydrogenase; CRP = C‐reactive protein; ESR = erythrocyte sedimentation rate.

The relationship between the tools and muscle disease measures and inflammatory markers was investigated (Table 3). DAS skin and CAT activity scores were both moderately inversely correlated with both CMAS and MMT8 scores. Inverse correlations were expected, given that muscle scores are low in severe weakness, while skin scores are high in the presence of high skin disease activity. However, this correlation was only moderate, reflecting the fact that skin disease can be active after myositis resolves and vice versa. MITAX skin was also inversely correlated with the MMT8 score, but no significant correlation was observed with the CMAS. There were no significant correlations between the muscle enzymes CK, ALT, AST, or LDH and any of the tools or their skin subsections. No significant correlations were found between the skin tools and inflammatory markers.

#### Estimation of disease activity

Having found that skin VAS correlated most strongly with each of the other tools, we next evaluated the capacity of each tool to predict skin VAS. Results of univariate and bivariate regression analyses to investigate the ability of the skin tools to accurately estimate skin disease activity, as measured by skin VAS, are summarized in Table 4. In univariate models, although all the measures were significant skin disease activity determinants, the skin sections of the tools were stronger than the global tools. The DAS skin appeared to be the strongest tool to evaluate skin VAS, based on having the highest model‐adjusted R^2^, therefore accounting for the greatest degree of variance in skin VAS.

**Table 4:** Linear regression analyses of the ability of the tools to estimate physician skin disease activity^a^

Predictor variable	Standardized parameter estimate	*P*	Model adjusted R^2^	ANOVA (F)	*P*	VIF
Univariate						
CAT activity	0.529	< 0.001	0.326	–	–	–
DAS total	0.268	< 0.001	0.335	–	–	–
DAS skin	0.633	< 0.001	0.521	–	–	–
MITAX global	0.124	< 0.001	0.148	–	–	–
MITAX skin	0.446	< 0.001	0.289	–	–	–
VAS global	0.492	< 0.001	0.318	–	–	–
Bivariate						
CAT activity	0.354	< 0.001	0.422	12.205	< 0.001	1.36
VAS global	0.322	< 0.001				
DAS total	0.165	0.01	0.370	4.732	0.03	2.19
VAS global	0.263	0.03				
DAS skin	0.515	< 0.001	0.557	6.539	0.01	1.45
VAS global	0.210	0.012				
MITAX total	0.064	0.230	0.323	18.775	< 0.001	3.04
VAS global	0.638	< 0.001				
MITAX skin	0.275	0.003	0.392	12.58	< 0.001	1.40
VAS global	0.337	< 0.001				

a ANOVA (F) = analysis of variance bivariate model compared to nested univariate model; VIF = variance inflation factor; CAT = Cutaneous Assessment Tool; DAS = Disease Activity Score; MITAX = Myositis Intention to Treat Activity Index; VAS = visual analog scale.

In the bivariate models of skin VAS, the addition of the physician's global VAS to the tools CAT activity, DAS total, DAS skin, MITAX global, and MITAX skin strengthened each of those models, as revealed by the ANOVA comparisons of the bivariate models to their respective nested univariate models (Table 4). Both variables in the bivariate model consisting of CAT activity and physician's global VAS were statistically significant, accounting for a proportion of variance in skin VAS of R^2^ = 0.422. Although the bivariate model using the combination of DAS skin and physician's global VAS appeared to be a stronger estimator of skin VAS (R^2^ = 0.557), the physician's global VAS was not statistically significant in this model (*P* = 0.012), using the stringent threshold of 0.001 (to allow for multiple comparisons). VIF values for each of the bivariate models indicated that multicollinearity was not likely to have affected the precision of the estimates.

For the 20 patients who were assessed at 2 separate time points, the SRM value for skin VAS, DAS skin, and CAT was 1.18, 0.55, and 0.15, respectively. Skin VAS displayed the greatest magnitude of change over time, skin DAS was moderate, and CAT was trivial. The intraclass correlation coefficients were all above 0.75, indicating excellent interrater reliability between the 2 physicians.

#### Time for instrument completion

Physicians individually kept track of their time to complete (i.e., to fill out the form and obtain a score) the skin section of each tool (MITAX, DAS, and CAT). The mean ± SD times to complete the forms for the MITAX skin, DAS skin, and CAT were 1.4 ± 0.24 minutes, 0.68 ± 0.1 minutes, and 0.63 ± 0.1 minutes respectively (n = 33 patients).

## DISCUSSION

Muscle disease is often the dominant clinical feature of juvenile DM. However, cutaneous involvement is a defining feature of juvenile DM and is an equally important manifestation of both disease activity and disease damage 17 as a source of morbidity and association with poorer outcomes 18, 19. For these reasons, skin disease activity and damage are essential components of the overall assessment of children with juvenile DM.

In this study we compared the 3 scoring tools to assess skin in juvenile DM patients. The CAT and both the skin sections of DAS and MITAX correlate well with the skin VAS. All 3 tools had moderate correlation with the physician's global VAS. Correlation with CK, ALT, AST, or LDH was not found. In addition, no significant correlations were found between the skin tools and the inflammatory markers, CRP level and ESR. The global MITAX and global DAS (2 tools that assess the skin as part of a generalized activity tool) had a higher correlation with the physician's global VAS than the skin VAS.

When linear regression models were used to estimate skin disease activity, the DAS skin, MITAX skin, and CAT activity were all significant estimators of skin disease activity. The addition of CAT activity in a bivariate disease estimator model containing the physician's global VAS was a statistically significant estimator of skin VAS score. This finding suggests that CAT activity provides additional information to the physician's global VAS, contributing to the assessment of skin disease activity. According to Cohen's threshold 15, the skin VAS showed the largest responsiveness to change. Although the other tools had lower SRM values, indicating less sensitivity to change, they were still highly correlated with skin VAS.

To our knowledge our study is the first of its kind among patients in a juvenile dermatomyositis population. A previous study compared 3 skin‐specific tools, the Cutaneous Dermatomyositis Disease Area and Severity Index (CDASI), the Dermatomyositis Skin Severity Index, and the CAT 20. In that study, 10 dermatologists used these instruments to score the same 12–16 adult patients with DM in 1 session. The overall disease activity at the time of the study was defined by the dermatologists completing a global physician score, while the patients completed a global patient score and global itch score. The CDASI was found to be a useful outcome measure for studies of cutaneous DM. However, these tools have not yet been validated in a pediatric population. The disease course differs from adults, as do the skin disease manifestations. Despite these differences, it would be interesting to test the use of the CDASI in pediatric patients in a future study.

One limitation of this study is that relatively few patients had very active skin disease; most had mild to moderate skin disease. This finding is likely to reflect the spectrum seen in most rheumatology services. However, since 61% of patients had a DAS skin score >4 (maximum score 9), we believe that the population had enough skin disease to allow testing of the tools. Another limitation of the study is that currently there is no gold standard to assess skin disease in juvenile DM. We found that both the physician's global VAS and the skin VAS correlated with all 3 tools, but the correlations with the skin VAS were higher. In a recent study analyzing detailed data from a subgroup of patients in the rituximab myositis trial, the physician's skin VAS was found to be sensitive to change in skin disease, with an SRM of 1.1 (*P* < 0.03) 21. We therefore chose to use the skin VAS in our regression analyses, because although skin VAS and overall VAS correlated well with each other (r_s_ = 0.75, *P* < 0.0001, data not shown) skin VAS correlated better than the physician's global VAS with the other skin tools.

Skin disease can be active after the myositis has resolved, and our own data demonstrate children with skin disease yet relatively low overall VAS scores, and vice versa. The routine and reliable measurement of skin activity in clinical practice could influence treatment choices in juvenile DM. Physicians should consider and document skin disease independently of muscle disease in the assessment of children with juvenile DM. This study represents an important step in the process of developing indicators to monitor skin disease activity in children with juvenile DM.

The study results demonstrate that all 3 tools effectively assess skin disease in children with juvenile DM. In a univariate model the correlation was strongest between the DAS skin and the physician's skin VAS. In a bivariate model, the addition of CAT to the physician's global VAS was the only one that maintained significance. However, all 3 tools have limitations that must be taken into account. The skin tools objectively capture items, but the weighting of the items varies between tools. The skin VAS can capture items not in tools, allows physicians to apply their own weighting, and allows skin activity to be assessed longitudinally in the clinic as well as for research. However, we propose that the use of a skin VAS alone as a single tool may miss important information, since individual skin features would not be documented.

We therefore recognize that there is scope for a new skin tool for juvenile DM that takes into account the strengths of the 3 existing tools, to be devised, tested, and validated in large cohorts using robust and internationally agreed standards 22. Until that time we propose the use of a skin VAS and one of these tools in routine practice could improve recognition and prompt treatment of ongoing skin disease in juvenile DM.

## AUTHOR CONTRIBUTIONS

All authors were involved in drafting the article or revising it critically for important intellectual content, and all authors approved the final version to be submitted for publication. Dr. Wedderburn had full access to all of the data in the study and takes responsibility for the integrity of the data and the accuracy of the data analysis.

**Study conception and design**. Campanilho‐Marques, Almeida, Nistala, Pilkington, Wedderburn.

**Acquisition of data**. Campanilho‐Marques, Almeida, Arnold.

**Analysis and interpretation of data**. Campanilho‐Marques, Almeida, Deakin, Gallot, Iorio, Nistala, Pilkington, Wedderburn.
